# Editorial: Development and Application of Molecular Technologies for Monitoring Microbial Contaminants in Water

**DOI:** 10.3389/fmicb.2021.671964

**Published:** 2021-07-27

**Authors:** Jingrang Lu, Yiping Cao, Hodon Ryu, Keya Sen, Eric N. Villegas

**Affiliations:** ^1^Center for Environmental Measurement & Modeling, United States Environmental Protection Agency, Cincinnati, OH, United States; ^2^Santa Ana Regional Water Quality Control Board, California Environmental Protection Agency, Riverside, CA, United States; ^3^Center for Environmental Solutions and Emergency Response, United States Environmental Protection Agency, Cincinnati, OH, United States; ^4^Division of Biological Sciences, STEM, University of Washington, Bothell, WA, United States

**Keywords:** molecular technology, monitoring, microbial contaminant, water, editorial

Microbial contaminants in waters, including viral, bacterial, and protozoan pathogens, continue to be a significant global public health concern. Detecting and predicting the presence and abundance of these microbial contaminants are critical to manage their health risks. Traditionally, monitoring of microbial contaminants has relied on microbial growth-based methods. However, advancements in molecular research and novel engineering processes have led to technologies for fast, specific, comprehensive, and high-throughput monitoring of microbial contaminants. This special issue focuses on the current state of development and application of molecular technologies and their implications for microbial water quality monitoring globally. This includes four original research articles and one review describing technological advancements and their applications that directly address issues related to microbial contamination of the global hydrosphere. Article contributions also range from industry, academia to government research institutes.

The first article, “Hepatitis E Virus (HEV) Occurrence in Waste and Drinking Water Treatment Plants” by Cuevas-Ferrando et al., focuses on monitoring the occurrence of HEV in influent and effluent waters in waste- and drinking-water treatment plants. This paper evaluates different procedures like aluminum hydroxide treatment and ultracentrifugation to concentrate and isolate HEV particles. HEV in influent and effluent water samples can be detected using the concentration technique together with aluminum hydroxide. HEV in drinking water samples can also be efficiently recovered using a primary ultracentrifugation method followed by a PEG secondary concentration. The performance of the selected concentration methods, coupled with two RNA extraction kits and three RT-qPCR assays, for detecting HEV in the influent and effluent water samples from four different wastewater treatment plants over 1 year is described. Overall, the recovery of HEV is impacted by not only the HEV concentration methods, but also the extraction kits and the RT-qPCR assays.

The second article, “An Optimized Method to Assess Viable *Escherichia coli* O157:H7 in Agricultural Soil Using Combined Propidium Monoazide Staining and Quantitative PCR” by Fu et al., describes the development of a propidium monoazide (PMA) qPCR method for the detection and quantification of viable *E. coli* O157:H7 in agricultural soil. The PMA-qPCR method coupled with a culture method is also utilized to quantify viable but non-culturable *E. coli* O157:H7, which typically cannot be determined by culture-based methods alone. When this optimized PMA-qPCR method is applied to both agricultural and paddy soils, the results demonstrate the PMA-qPCR to be a more sensitive and rapid approach for quantifying viable *E. coli* O157:H7 than the culture methods.

The third article, “Monitoring of Nitrification in Chloraminated Drinking Water Distribution Systems with Microbiome Bioindicators Using Supervised Machine Learning” by Gomez-Alvarez and Revetta, describes an approach that uses microbiome-based bioindicators to predict operational failures in drinking water distribution systems (DWDS) (e.g., nitrification). These bioindicators, which describe the microbial populations responsible for inorganic nitrogen-compounds (e.g., NH_3_, ${\text{NO}}_{2}^{-}$, and ${\text{NO}}_{3}^{-}$) during onset of nitrification, may reveal the potential onset of a nitrification event before sufficient levels of these nitrogen-compounds are detected. This approach is different from traditional culture-based methods for estimating the most probable number of ammonia oxidizing bacteria (AOB), which require weeks of incubation and lack the ability to detect low levels of AOB in the DWDS. Although this microbiome-based bioindicator approach still requires additional validation before use as a standard method, and is currently limited as a screening tool, the approach has tremendous potential for predicting nitrification in DWDS.

The fourth article, “The Bacterial Community Diversity of Bathroom Hot Tap Water was Significantly Lower than That of Cold Tap and Shower Water” by Zhang et al., describes 16S rRNA sequence-based microbial community comparisons among cold tap, hot tap, and shower water of a bathroom premise plumbing system (PPS) over a year. The major findings are that (1) five groups (*Burkholderiaceae, Sphingomonadaceae, Alphaproteobacteria, Corynebacteriales*, and *Mycobacteriaceae*) dominate PPS microbial communities, (2) the bacterial community structure of hot tap water is significantly different from those of cold tap and shower water, (3) the bacterial community diversity is lower in hot tap water than in cold tap and shower water, and (4) the water temperature, total chlorine residual concentration, stagnant status, and the age of the home plumbing system simulator impact bacterial community diversity. Collectively, microbial communities tend to be more complex and numerous in cold tap than in hot tap, where high-temperature resistant populations are dominant.

The fifth article, “Aptamers and Aptamer-coupled Biosensors to Detect Water-borne Pathogens” by Saad and Faucher, is a review article that focuses on the use of aptamers as efficient bioreceptors for the development of biosensing detection platforms. They describe several recently developed aptamer-based detection systems used on key water-borne pathogens like norovirus, *Giardia duodenalis* cysts, *Acinetobacter, Aeromonas, Campylobacter*, cyanobacteria, *Escherichia coli, Helicobacter pylori, Legionella*, non-tuberculous mycobacteria, *Pseudomonas aeruginosa, Shigella, Vibrio cholera*, and *Yersinia*. Successful applications of aptamers include those targeting microbes in specific states and growth conditions (e.g., *Cryptosporidium* oocyst, starved *L. pneumophila* and biofilm-derived *Pseudomonas aeruginosa*), targeting viable cells (e.g., *S. enterica* serovar Typhimurium*, C. jejuni*), and targeting specific surface structures (e.g., whole cell *S. enterica* Typhimurium and lipopolysaccharides of *E. coli* O157:H7). There are still challenges for aptamer's application as officially adopted methods, although aptamer-coupled biosensors are promising systems for the detection of pathogens in water samples. A collaborative effort between academics and stakeholders is needed to develop both transducer and aptamer technologies for specific microbial contaminants.

This collection of articles is just a sample of the various molecular-based technologies that are available and their potential application in future water quality monitoring efforts. More importantly, this special issue reveals the challenges ahead with standardizing and implementing these methods for routine monitoring. Most current work requires highly specialized training and are performed by various research laboratories, which likely follow different protocols. Wide application with consistent performances among the laboratories, in turn, are fundamental to efficient and effective water quality monitoring programs. For example, to take these technologies beyond research and apply them toward supporting routine water quality monitoring program requires significant investments in infrastructure, resources, and technical staff prior to implementing the method. Technical staff training, technology transfer, method standardization, and interlaboratory performance comparison are only a few required efforts needed. Indeed, tools being used for microbial source tracking, the global microbiome project, the eDNA barcoding, and most recently, the qPCR assays used for monitoring SARS-CoV-2 (the virus causing COVID-19) in wastewater are prime examples of molecular-based tools that are currently undergoing method standardization and technology transfer, through interlaboratory comparison and round robin studies. Laboratory accreditation to conduct these molecular-based techniques for environmental monitoring is still in its infancy, and should look toward clinical diagnostics as a “model” for implementing these technologies for routine monitoring efforts on environmental samples. Future development on automation (including both instrumentation and informatics pipeline) would also play an important role in realizing the potential of molecular technologies for water quality monitoring.

## Author Contributions

JL has defined the aim and scope of the Special Issue, assisted in inviting contributions, been the final decision-maker for articles after peer-review, and collaborated with his editorial team at Frontiers in Microbiology. The associate editor YC, HR, KS, and EV have assisted in revising the aim and scope of the Special Issue and inviting contributions, and have been the final decision-maker for articles after peer-review. All authors contributed to the article and approved the submitted version.

## Author Disclaimer

The U.S. Environmental Protection Agency through the Office of Research and Development did not collect, generate, evaluate, or use the environmental data described herein. The views expressed in this manuscript are those of the authors and not necessarily reflect on the Agency. It has been subjected to Agency review and approved for publication. Mention of trade names or commercial products does not constitute endorsement or recommendation for use.

## Conflict of Interest

The authors declare that the research was conducted in the absence of any commercial or financial relationships that could be construed as a potential conflict of interest.

## Publisher's Note

All claims expressed in this article are solely those of the authors and do not necessarily represent those of their affiliated organizations, or those of the publisher, the editors and the reviewers. Any product that may be evaluated in this article, or claim that may be made by its manufacturer, is not guaranteed or endorsed by the publisher.

